# Crustal structure in the Campanian region (Southern Apennines, Italy) from potential field modelling

**DOI:** 10.1038/s41598-021-93945-8

**Published:** 2021-07-15

**Authors:** Y. Kelemework, M. Milano, M. La Manna, G. de Alteriis, M. Iorio, M. Fedi

**Affiliations:** 1 University of Naples Federico II, DiSTAR, Naples, Italy; 2 Institute of Marine Sciences, National Research Council of Italy (CNR–ISMAR), Naples, Italy

**Keywords:** Structural geology, Geophysics

## Abstract

We present a 3D model of the main crustal boundaries beneath the Campanian region and the onshore and offshore surrounding areas, based on high-resolution potential field data. Our main objective is the definition of the main structural interfaces in the whole Campanian region from gravity and magnetic data, thanks to their ability to define them on a regional and continuous way. The complex morphology of the Mesozoic carbonate platform, which is fundamental to constrain the top of geothermal reservoir, was reconstructed by inverting the vertical gradient of gravity. We assumed local information from seismic models and boreholes to improve the model. We modeled the deep crustal structures by spectral analysis of Bouguer gravity and magnetic data. The inferred depth estimates indicate a shallow crystalline basement below the Tyrrhenian crust and the Apulian foreland and a significant depression beneath the Bradanic foredeep. The map of the Moho boundary shows a NE-SE verging trough below the Southern Apennine chain and two pronounced uplifts beneath the foreland and the Tyrrhenian crust. We also estimated the depth to the magnetic bottom, showing a thick magnetic crust below the mountain chain and shallow depths where the crustal heat flow is high. The models were compared with seismic sections along selected profiles; a good agreement was observed, despite of some inherent lower resolution for the gravity modelling from spectral methods. The regional covering and the continuity of our estimated crustal interfaces make it a new and valid reference for further geological, geophysical and geothermal studies, especially in areas such as northern and eastern Campania, where there is an incomplete geophysical and geological information.

## Introduction

The Apennine chain, linking the western Alps to the Maghrebian orogen, is one of the main orogenic belts of the central Mediterranean, resulting from the collisional events between the African and the European plates, which took place since the late Mesozoic-Cenozoic Alpine orogeny^1–3^. Special interest was reserved, in last decades, to the Southern Apennines, where intensive hydrocarbon exploration and geothermal potential exploitation made available numerous geophysical data spanning from well logs to seismic and potential fields^4–13^. As a result, geological models of the shallow and deep crustal architecture have been proposed by integrating seismic data with structural, stratigraphic and borehole data. However, seismic data as well as potential field data can be affected by errors and uncertainties and to make reliable models of deep structures, such as the crystalline basement and Moho surface^14,15^ we need to integrate seismic, wells and potential field data. This is especially true along complex geological environments, where seismic reflection imaging and well data are often reliable only to a depth of about 10 km^4,16^. As for potential field data, they include the effect of multiple crustal sources and the inherent non-uniqueness of the inverse problem requires a-priori information to produce reasonable results. The best advantage of potential field modelling with respect to seismic is ensuring a wide areal coverage, so yielding lateral continuity to the subsurface models^17^.

Within this framework, the purpose of this paper is providing new models of the Mesozoic carbonate platform, crystalline basement, Moho boundary and magnetic bottom beneath the Southern Apennines orogen. It is a simplified model, not accounting for more complex source heterogeneities in the crust, so that they are valid on a regional scale but may fail locally, as in the volcanic regions.

## Geological setting

The intricate geological framework of the Italian peninsula is the result of several geodynamic events associated to the opening and closure of the Tethyan ocean. The Apennine orogen is a Neogene and Quaternary thrust belt developed during the Africa-Europe collision and consisting of an accretionary wedge and a back-arc Tyrrhenian basin to the west, which is progressively migrating eastward since the Miocene^3,18–25^. To the East, the Adria plate represents the foredeep and foreland region of the orogenic belt^26–28^. The northern Apennines is characterized by a regular, in-sequence system of N and NE-verging thrust imbricates^3^, while the Southern Apennines consist of ENE and E-verging thrusts which possesses duplex geometries and out-of-sequence trusting^1,3,29,30^. The central Apennines display N-verging and NE to ENE-verging thrust faults that dissect the tectonic edifice into several, small-scale tectonic slices^3,31^. The Tyrrhenian Sea-Apennines system is a well-paired tectonic belt with shortening on the foreland side of the orogen and extension in the hinterland^29,32,33^.

The extensional Tyrrhenian basin located between Sardinia, Sicily and Peninsular Italy is characterized by partly oceanic and thinned continental crust with an irregular seafloor^2,3,22,23,33,34^. It represents a back-arc extensional feature developed at the rear of the Apennine system in late- and post-Tortonian times. The peri-Tyrrhenian margin has undergone extensional tectonics during Plio-Quaternary accompanied by magma up rise and partly still active volcanism recognizable both at sea and onshore (see^35^ for a comprehensive review).

**Figure 1:**
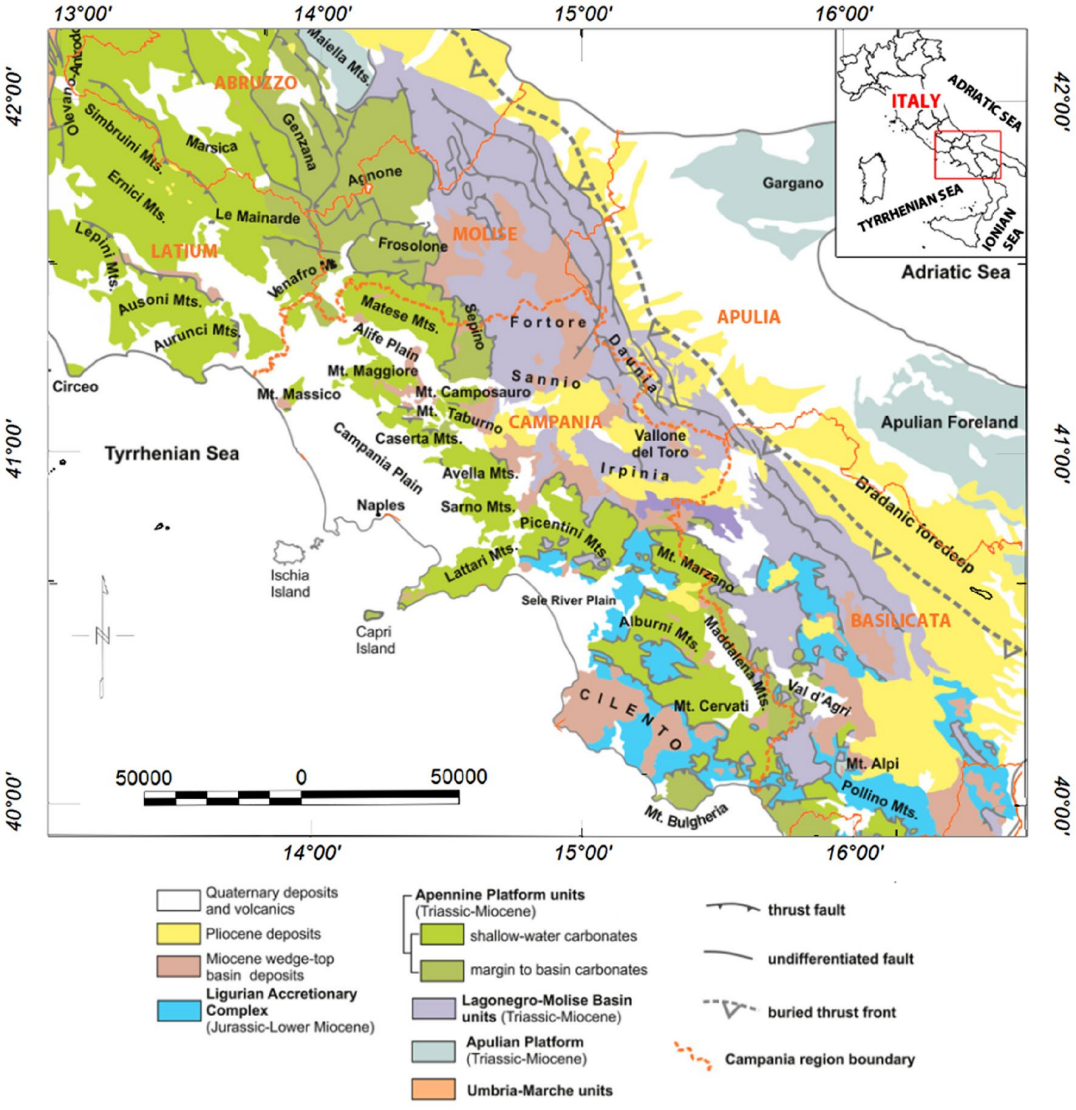
Sketch geological map of the Southern Apennines (modified after^36^). The map was generated using the Oasis Montaj Geosoft software (v.9.9.1.34 https://www.seequent.com/products-solutions/geosoft-oasis-montaj/).

The entire Apennine fold-and-thrust belt is made up of several main litho-stratigraphic units (Fig. 1) that can be correlated to the following paleogeographic domains (see^23,36^ for a complete review):

1. The Adriatic-Apulia carbonate platform represents the pre-orogenic cover of the foreland area, composed of Mesozoic-Tertiary carbonates and Triassic evaporites overlying a thick pile of mixed carbonate-siliciclastic Paleozoic deposits^2,3,23,26^. The Adriatic-Apulia carbonate units are deep beneath the Campania region while they are outcropping in the eastern sector of the Puglia region (Gargano, Murge, and Salento) and overly a Permian or Ladinian-Carnian volcanoclastic or sedimentary basement^23,36^.
2. The Lagonegro-Molise basin consists of Meso-Cenozoic shallow-water to pelagic sediments. The stratigraphic succession is represented by fluvial conglomerates and shallow water siliciclastic deposits followed upward by pelagic cherty limestones cherts and radiolarites and finally by silicified marls and clays (^23,37^ and references there in)
3. The Apennine carbonate platform, also known as the Campano-Lucana platform, consists of shallow water and subordinate deeper-water Mesozoic-Tertiary carbonates formed in peritidal and lagoonal environments and platform-edge and slope facies^23,37^. It is partially overlain by a thick pile of Plio-Pleistocene, syn-orogenic and post-orogenic deposits^2,3^.
4. The Liguride and Sicilide units comprises of both metamorphic unmetamorphosed sediments of early Cretaceous to early Miocene age located internally to the Apennine orogen^23,37,38^.

Crustal subduction dynamics led to the formation of extended depressional areas such as the Bradanic Trough, filled by deep to shallow marine deposits^39^. The internal nappes comprise of sediments derived from internal domains scraped off from the subducted crust of the Ligurian-Piedmont Neotethyan Ocean and includes the Liguride units and Sicilide units (^23,37^ and references therein).

## Geophysical background

In the last few decades several geophysical studies have been conducted to understand the deep crustal structure of the Italian Peninsula.

Among several geological and geophysical projects, VIGOR (‘Valutazione del potenzIale Geotermico delle RegiOni della convergenza’^40^) provided an opportunity to assess the regional geothermal potential in Campania and southern Italy^13,41–44^.

Models of the crust-mantle transition have been carried out^45–50^ through wide-angle refraction profiles (DSS)^51–56^, Bouguer gravity data^57–60^ and CROP deep seismic reflection surveys^61–64^. The Moho topography beneath Europe and the Mediterranean region has been mapped since the 1960s^64–67^, with the huge CROP project being the most significant advancement in the interpretation of the crustal structures of the Italian Peninsula^61^. A rich literature was indeed recently produced, based on the interpretation of the CROP seismic transects, aiming at unveiling the anatomy of the Apennine orogen and the surrounding regions^61,64,68^. Overall, the Apennines are characterized by a deep seismic Moho depth that varies from more than 40 km to the north to about 35 km in the central and Southern Apennines^45,69,70^. The Tyrrhenian Sea is characterized by a relatively thin crust (20–25 km) in west Tuscany and Latium, reaching 10 km in the southeast Tyrrhenian Sea, beneath the Vavilov and Marsili basins, separated by the Issel bridge (15 km)^45,69–71^. In the stable regions (Adriatic Sea and Apulia) the Moho depth is estimated at about 30 km.

The study of the gravity and magnetic anomalies^64,71–74^ contributed to extend and complete the geological interpretation referred to other sparse geophysical data, such as seismic and well logs. Some authors proposed a composite internal wedge of the high-susceptibility lower crust beneath the Southern Apennines, so explaining the presence of intense magnetic anomalies parallel to the trend of the orogen. Gravity data modeling^74–77^ also suggested a ramp-dominated style of the lower crust, involving the crystalline basement, which has been considered as the main source of the observed regional-scale gravity anomalies. However, the nature of the crystalline basement beneath southern Italy and its degree of involvement into thrusting and shortening remains matter of speculation and debates.

Temperature and heat flow data have been collected, compiled, and presented in the form of anomaly maps and profiles for the Italian peninsula and surrounding regions^78^. Heat-flow values are generally low (40–60 mW/m^2^) in the Mesozoic–Cenozoic carbonate units of the Southern Apennine fold and thrust belt and decrease to 20–40 mW/m^2^ in the central Apennines. Heat flow is very high (up to 200 mW/m^2^ or more) in the Tyrrhenian Sea and Western Apennines, particularly in Tuscany, while its decreases to 30–40 mW/m^2^ in the foreland areas (Adriatic coast and Ionian Sea)^78^.

## Data and methods

**Figure 2:**
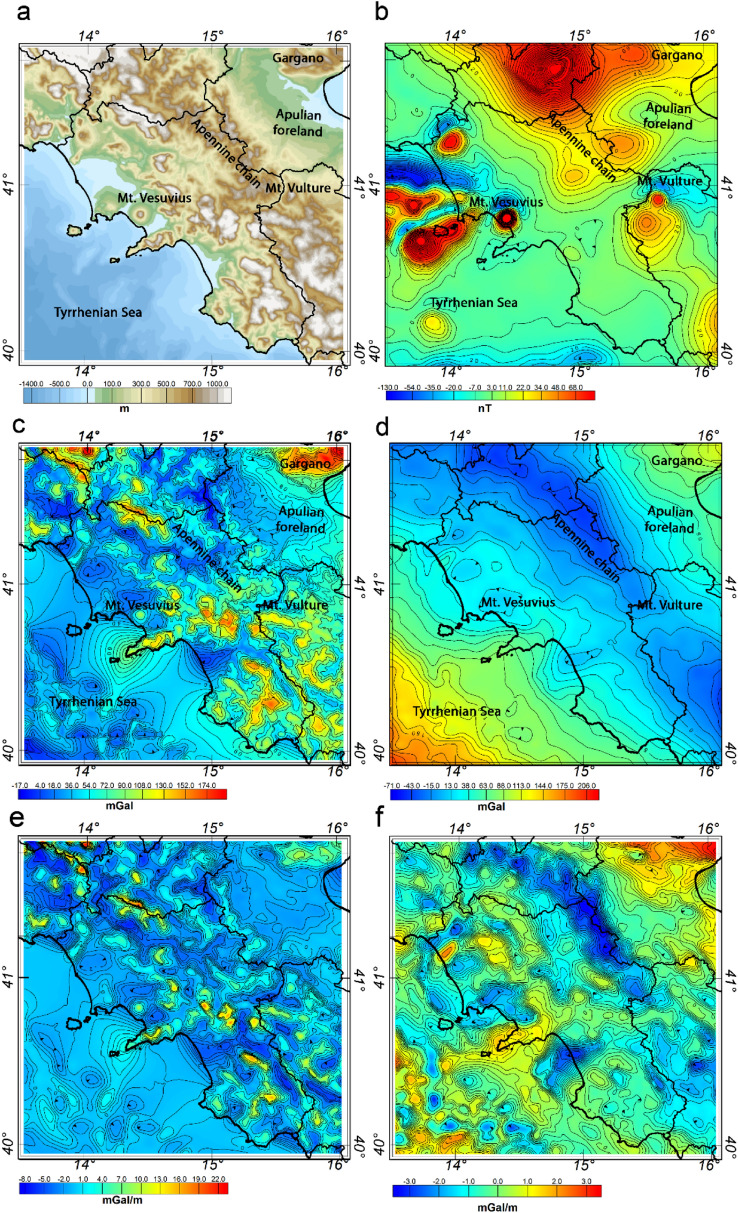
Maps of the topography of southern Italy (**a**); the aeromagnetic field map (**b**); Free-air (**a**) and Bouguer (**b**) gravity field maps and the vertical gradient of the Free-air (**c**) and Bouguer gravity field. The maps were generated using the Oasis Montaj Geosoft software (v.9.9.1.34 https://www.seequent.com/products-solutions/geosoft-oasis-montaj/).

To infer the crustal structure of Southern Apennines we used high-resolution gravity and magnetic field data. The aeromagnetic data (Fig. 2b) were compiled from different ground, onshore and offshore aeromagnetic surveys, mostly conducted during the 1970s and 1980s^79,80^. In this study, we merged and gridded all the data to a common projection at 4 km above mean sea level, with a 1 km sampling interval. The magnetic map of the Southern Apennine shows two main domains reflecting the different magnetic nature of western and eastern Italy. The Tyrrhenian region is characterized by short-wavelength anomalies, clearly associated to the diffuse presence of highly magnetized volcanic and magmatic rocks along the coast and in the Tyrrhenian Sea (^81^ and references therein). On the other hand, in the Apulian and Adriatic foreland we observe low amplitude anomalies along the external Apennine thrust and fold belt and the southern Adriatic Sea, which could be associated to the uplift of the magnetic basement and to crustal stretching and thinning events^81^.

The gravity data of the Southern Apennine and surrounding regions has been extracted from the Bouguer and Free-air gravity anomaly datasets of the Italian peninsula, merging the offshore gravity measurements with the onshore dataset (Fig. 2b). The normal gravity is based on the Geodetic Reference System 1980 (GRS80^82^) and projected with respect to the IGSN71 reference system, whereas Bouguer correction and terrain reductions were calculated using a 2.6 g/cm^3^ density reference and a digital elevation model of the topography and bathymetry with a 100 m resolution (Fig. 2a). Then, like the magnetic dataset, all the gravity data were gridded to a common projection and sampling interval of 1 km. The Free-air gravity field map (Fig. 2c) show a pattern of small-scale anomalies mostly associated to the complex topography of southern Italy, while the Bouguer gravity field (Fig. 2d) of the Southern Apennine shows two regions of positive anomalies in correspondence with the Tyrrhenian Sea and the Adriatic coast and a NW–SE verging trend of negative anomalies along the thrust front of the Apennine chain. There is a direct relationship between the main structural features and the large-scale Bouguer anomalies of the gravity field, especially above the thick deposit units of the foredeep.

In Fig. 2e,f we show the maps of the vertical gradient of the Free-air and Bouguer gravity data. The vertical gradient data are estimated by the ISVD method^83^, which is more stable than using the Fourier vertical derivative operator because it combines the vertical integration filter use (which is a smoothing filter) with the finite-differences method.

In particular, the carbonate top is mostly inferred by the vertical gradient data of the Free-air gravity anomalies (which are in practice related to the topography-related sources) and by applying a non-linear parameter estimation method. Differently, we interpreted the deep structural interfaces from spectral analysis of vertical gradient of the Bouguer gravity field and the aeromagnetic dataset. So, for the various interfaces we are dealing with different wavenumber contents and different anomaly fields.

We estimated the depth to the Mesozoic carbonates using the non-linear inverse method proposed by Fedi^84^, which consists of assuming an interface separating two media of different density, discretized into a set of homogeneous and adjacent prisms with variable depth to the top and thickness. An important feature of the method is that there is no need of a-priori information of the rock densities to solve the inverse problem. Instead, we used borehole and seismic information to constrain the morphology of the basement top. For a complete review and detail description of the theoretical formulation, we refer to the “Methods” section and to the original paper^84^.

As for the deepest crustal structures, there are different spectral techniques to get an independent estimate of regional crustal depths from potential field data. We used the statistical source model^85^ for the estimation of the depth to the top and the modified centroid method for the depth to the bottom estimate^86^. We used 80 × 80 km^2^ window size in the Tyrrhenian Sea where seismic study indicates an expected depth to the bottom of around 20 km and a 100 × 100 km^2^ window size in the Apennines area, where the expected depth to the bottom is around 30 km. We refer to “Methods” section for a detailed description of the spectral methods and the selection of the optimum window size, which is a critical issue.

**Figure 3:**
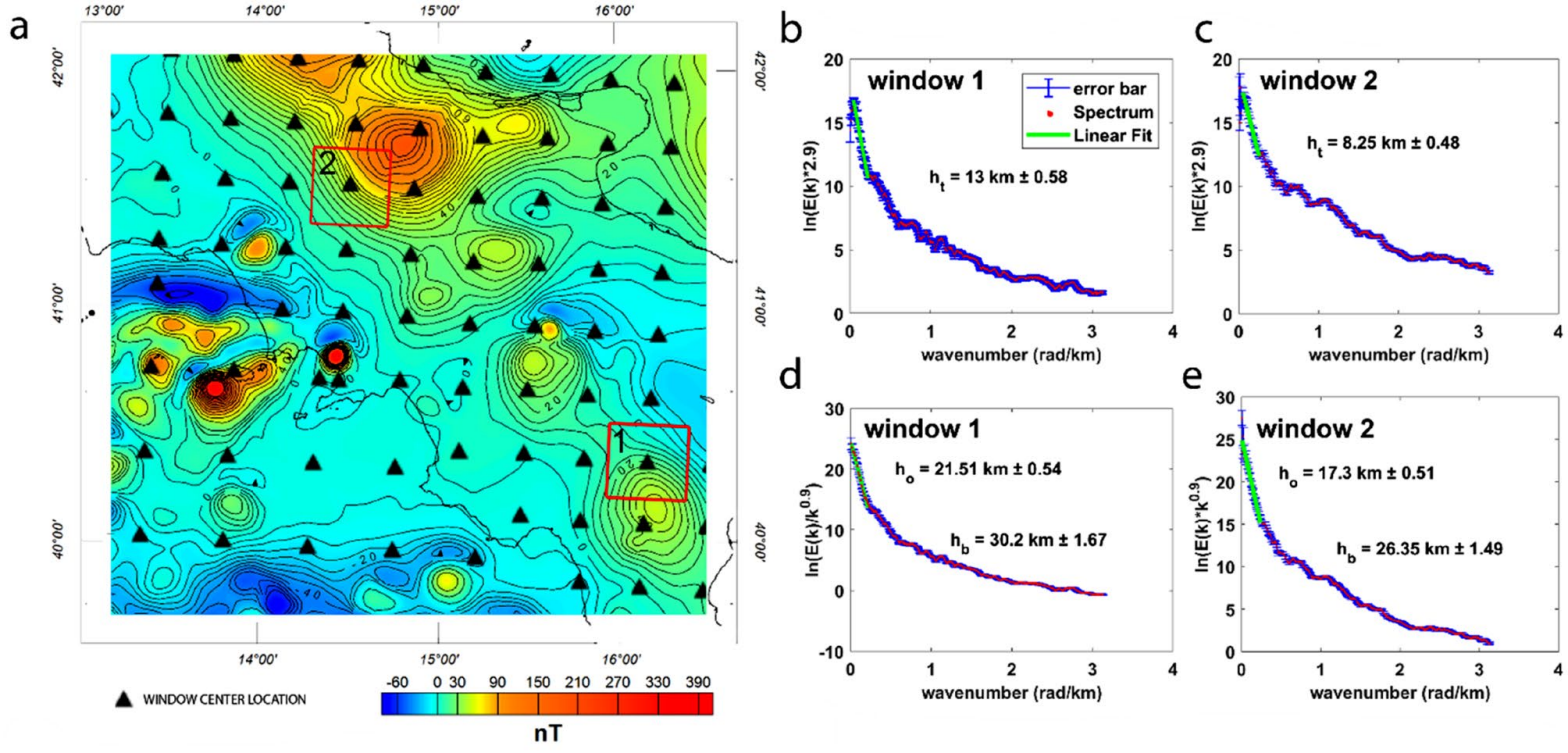
(**a**) Map of the aeromagnetic field in the Southern Italian region. Black triangles indicate the windows center used for the spectral analysis. We show examples of radially averaged power spectra of the magnetic field for estimating the depth to the top (**b**,**c**) after correcting the power spectra by *k*^−2.9^ and the radially averaged wavenumber-scaled power spectra for estimating the depth to the centroid (**d**,**e**) after correcting the power spectra by *k*^−2.9^. The location of windows is indicated by red squares (windows 1 and 2). *h*_*t*_ is the depth to the top, *h*_*o*_ the depth to the centroid and *h*_*b*_ the depth to the bottom, with their respective uncertainty.

The depths to the source top and centroid were estimated from the slope of the radially averaged power spectra and the slope of the radially averaged wavenumber-scaled power spectrum in each window, respectively [see “Methods”, Eqs. (9) and (11)]. The wavenumber range was chosen as that where the logarithm of the power spectrum is well approximating a straight line^85^ (Fig. 3b–e).

For the accuracy of depth estimates see “Methods” (Eqs. 13 and 14); error bars indicate the 95% confidence intervals for the radial average power spectrum computed within each ring (Fig. 3b–e). Finally, we note that the assumption of a simple interface-model may fail in the presence of local important heterogeneities, like volcanic or magmatic rocks, limiting our ability to define the right behavior of the interface.

## Results

### Modeling of the carbonate basement top

**Figure 4:**
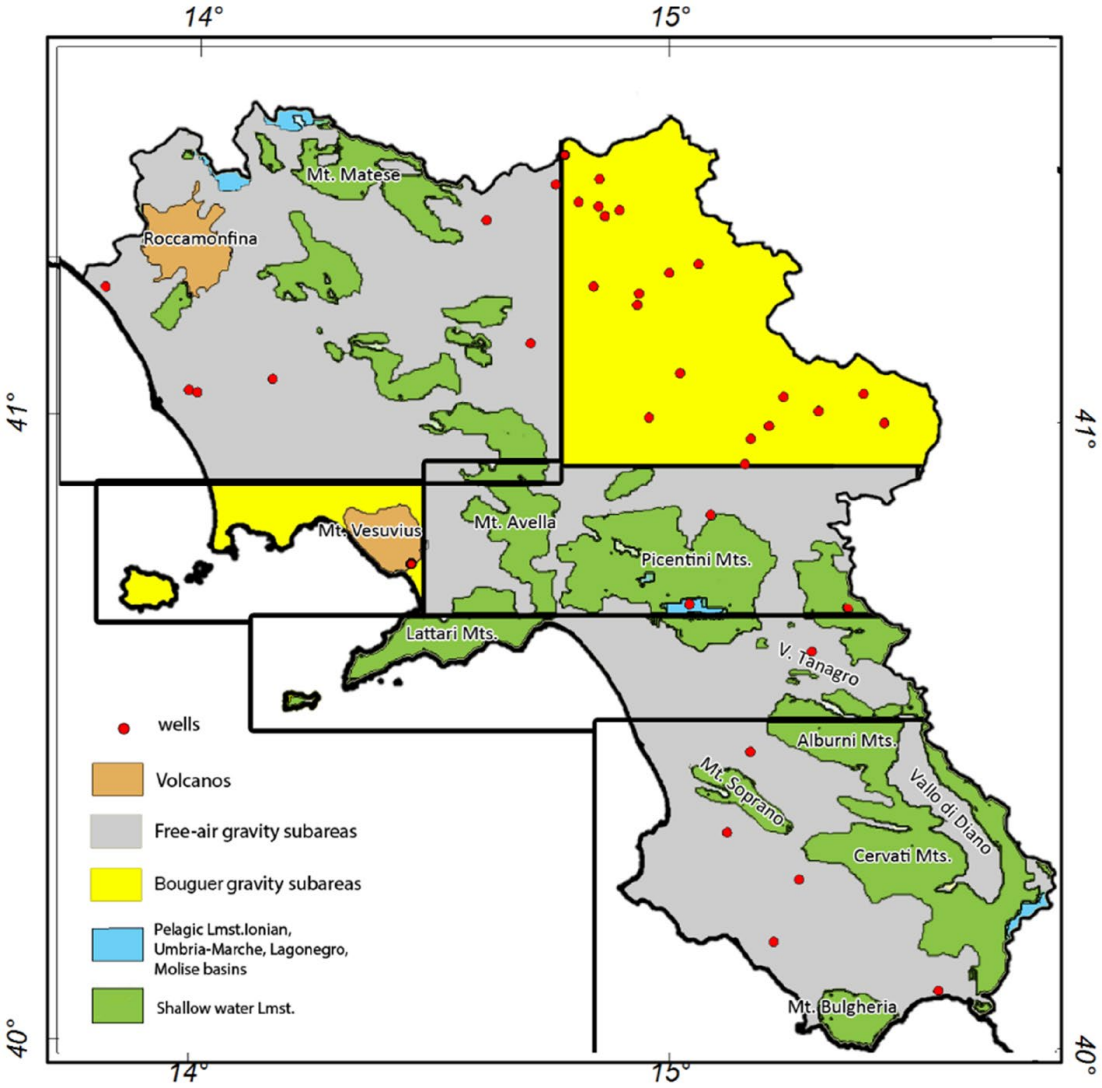
Map of the carbonate outcrops in the Campania region (modified after^28^). Black boxes represent the sub-areas selected to perform the inversion of free-air gravity data (gray color background) and of Bouguer gravity data (yellow color background).

A new map of the carbonate basement top of the Campania region is here presented, as result of the inversion of the vertical gravity gradient data in an area of around 22,000 km^2^ extent. Before performing inversion, we divided the study area into different subregions (Fig. 4), in order to isolate the areas of the carbonate outcrops, generally corresponding to the mountain reliefs, from those in which the carbonate units are assumed to be buried and deep. In fact, since the morphology of the carbonate basement is complex and mostly outcropping, we had to decide whether to use the Bouguer anomaly data or the free-air gravity field in each sub-area^17^. This is necessary, since the Bouguer slab reduction removes most of the gravity effect related to the topography. In almost the whole Campania region the carbonate is outcrop or it is at shallow depths, so that free air gravimetric anomalies are preferred (Fig. 4). Only in two small sub-areas (yellow colored in Fig. 4) we used the Bouguer vertical gradient anomalies, because the carbonates rocks are there overlaid by a stack of quaternary sediments units or volcanic rocks.

In each subarea we set at least two local constraints for the basement surface, deriving from available wells data, outcrop information or seismic interpretation (Progetto ViDEPI https://www.videpi.com/videpi/videpi.asp). Then, we inverted for the basement surface according to the method described in Fedi^84^. In some sub-areas more well constraints were used, and we ran more inversions using different combinations of them. Well-data yielding a poor data fitting were discarded.

**Figure 5:**
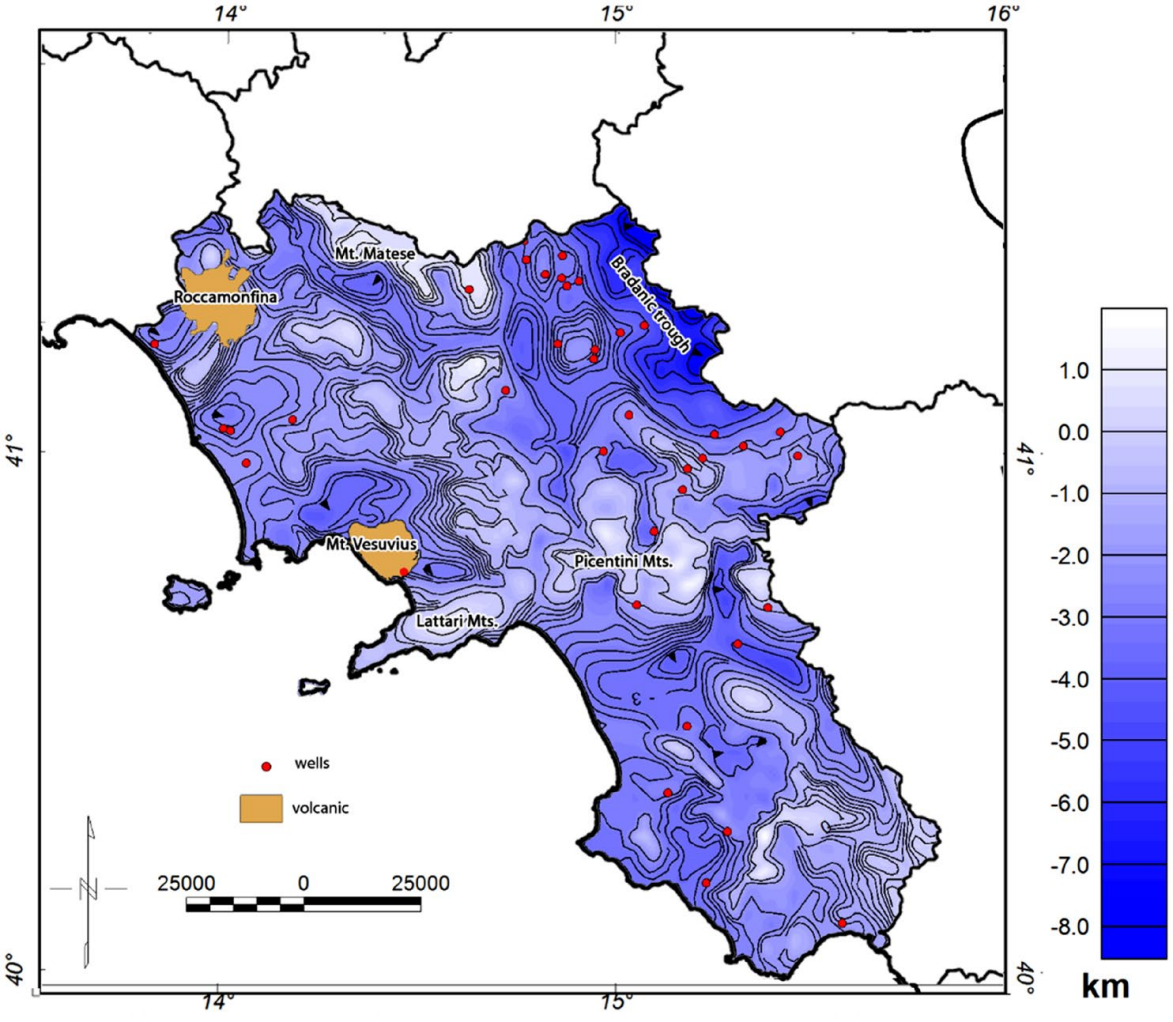
Model of the Mesozoic carbonate top of the Campania region obtained by the inversion of free-air and Bouguer gravity data.

The final map of the carbonate basement top was obtained by merging the results carried out in each subarea using the GridKnit function of the Oasis Montaj Geosoft software (Fig. 5). The resulting model shows a variable surface reaching a maximum depth at around 9 km beneath the Bradanic Trough, which is in good agreement with the whole outcropping carbonates, i.e. not only at the outcrop points used as constraints, such as above the Picentini Mts., Matese Mt. and the Lattari Mts. However, since the inferred depths may suffer from the presence of volcanic and magmatic rocks, we do not consider the results obtained beneath Mt. Vesuvius and Roccamonfina.

### Depth to crystalline basement

**Figure 6:**
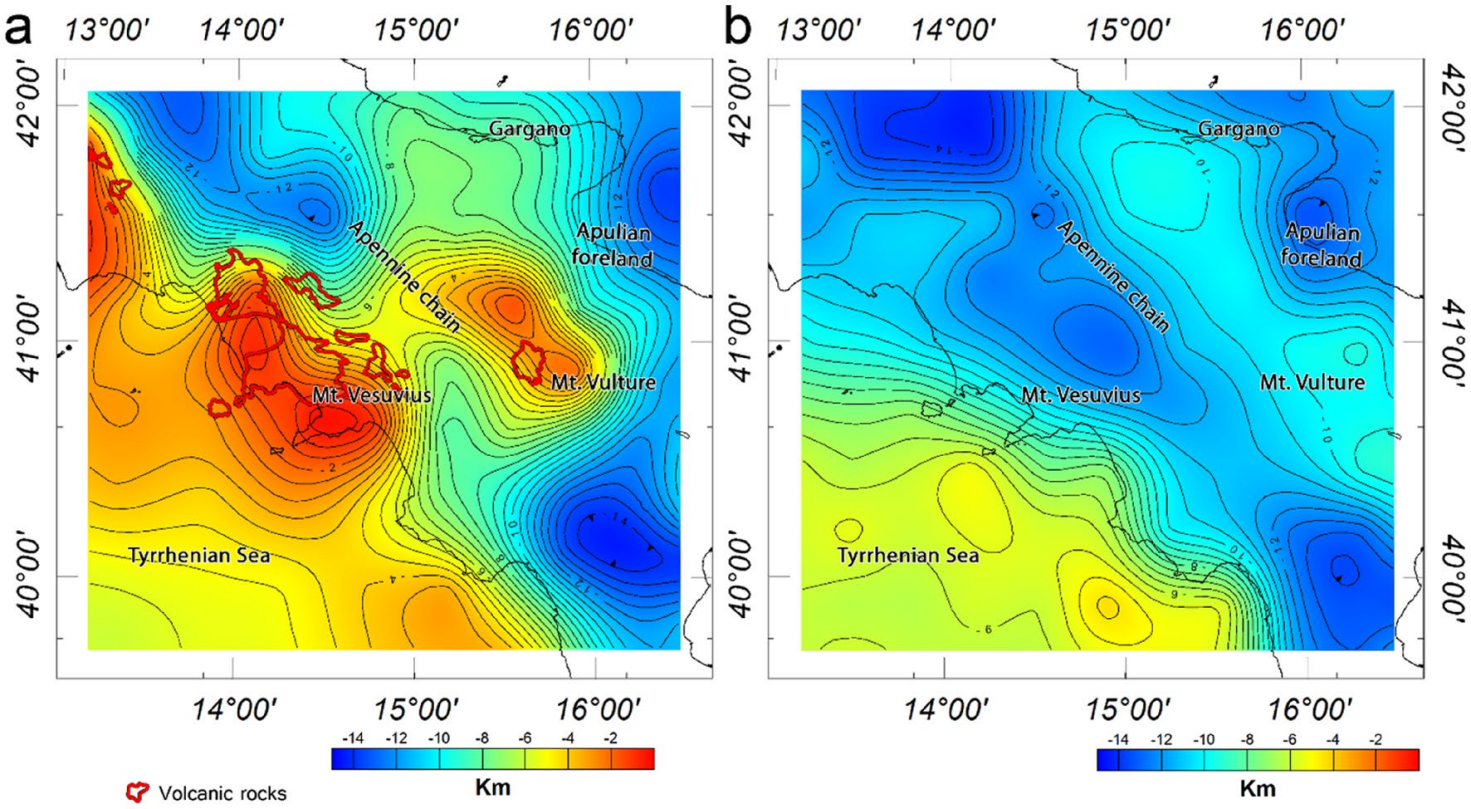
Depth to the top of magnetic sources estimated from magnetic anomalies (**a**) and depth to the top of crystalline basement estimated from the vertical gradient of gravity data (**b**). The maps were generated using the Oasis Montaj Geosoft software (v.9.9.1.34 https://www.seequent.com/products-solutions/geosoft-oasis-montaj/).

The map of the magnetic basement top is shown in Fig. 6a, where the average depth varies from less than 2 km in the Tyrrhenian region down to 14 km beneath the fold-and thrust-belt, the Calabrian arc, while it is around 12 km below the Apulian foreland. A shallow depth to the top of the magnetic sources is also observed beneath and around the Mount Vulture and along the Gulf of Naples. These shallow depths are most probably attributed to the volcanic rocks exposed to the surface or the existence of shallow magnetic sources. Magnetic basement depth values are estimated to be 10–13 km beneath the southern Adriatic Sea, which is slightly deeper than the basement values inferred beneath the Apulian platform. In fact, this Mesozoic-Cenozoic basin represents the foreland of the Apennine orogenic system to the west, the Dinaric to the east and the Alps to the north^15^, and shows a smooth basement morphology variation than the surrounding regions. We estimated a very deep (14 km) basement beneath the southern end of Apennines and the Calabrian arc, then gradually decreasing to less than 8 km toward the Ionian Sea and toward SE.

Note that the maximum depth of the magnetic sources might not be detected where there are extensive volcanic rocks on the surface or at shallow depths (i.e. Campania Volcanic Province, Roman Magmatic Province, and the Mt. Vulture). This limitation can be however well complemented by gravity data, which we find to be more suitable to model the crystalline basement top in regions affected by volcanism and intrusive bodies/dikes^17^.

The depth to the crystalline basement estimated from gravity data is shown in Fig. 6b. The inferred spectral depth estimates vary from about 4 to 6 km beneath the back-arc Tyrrhenian basin to 12 to 15 km beneath the Southern Apennines thrust and fold belts. A depth of about 15 km is found beneath central Apennines, progressively decreasing to about 11 km beneath Mount Vulture, and again increasing to about 14 km beneath the Calabrian accretionary prism.

The depth estimates for the regions underlying the Apulian foreland varies from 8 to 9 km. The depth decreases from about 10 km beneath the Gargano area to about 8 km in the southern end of the Apulian Platform. The depth to the crystalline basement morphology over the southern Adriatic Sea is more variable, from 13 km along the Apulian coastline to 10 km offshore.

### Depth to the bottom of magnetic sources and the Moho boundary

**Figure 7:**
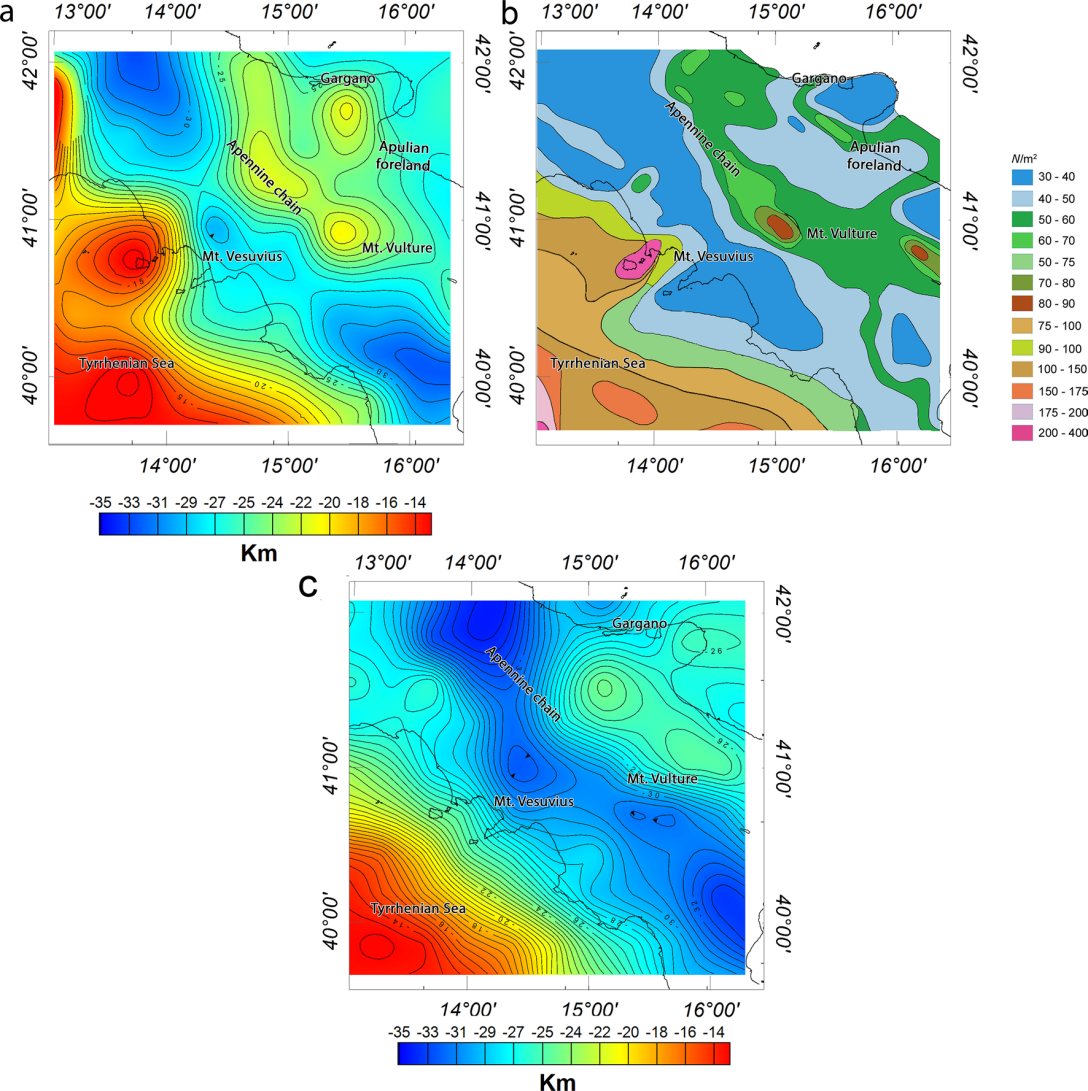
Depth to the bottom of magnetic sources (**a**), heat flow map (**b**) and Moho depth estimated from gravity data (**c**). The maps were generated using the Oasis Montaj Geosoft software (v.9.9.1.34 https://www.seequent.com/products-solutions/geosoft-oasis-montaj/).

Figure 7a,c show the depth to the bottom of magnetic sources (estimated from magnetic data) and the Moho boundary topography (estimated from gravity data), respectively. The depth to the bottom of magnetic sources often represents the thermal boundary of ~ 580 °C, a temperature beyond which rocks lose their magnetic properties. This is often referred to, somewhat improperly, as the Curie isothermal surface. Estimating the Curie isothermal depth is fundamental to establish the thermal setting of a region and to define the thickness of magnetic sources/layers.

So, we expect that the model of the depth to the bottom of magnetic sources mark the major thermal structures of the Southern Apennines and of the surrounding environs. We also show the map of the crustal heat flow which has been compiled from the data collected by^78,87,88^. A general comparison between the depths to the bottom of magnetic sources and the heat flow map (Fig. 7b) suggests that high heat-flow values are generally associated with shallow depth to the bottom of magnetic sources and vice-versa.

The depth to the bottom of magnetic sources varies between 12 km of depth beneath the Tyrrhenian crust to more than 34 km of depth beneath Southern Apennines. Specifically, the shallow depths to the magnetic bottom beneath the Tyrrhenian Sea and the northern coast of Campania corresponds to a very high heat flow, greater than 200 mW/m^2^^78^. The magnetic basal depth beneath the hanging wall of the Apennine varies from about 28 km in the southern Calabrian arc, deepening to 33 km southward and northward well corresponding with low heat flow ranging between 30 to 50 mw/m^2^. The depth to the bottom of magnetic sources is relatively shallow in the Mt. Vulture area, with a N-S direction, where high heat flow values occur (Fig. 7b, 80–90 mW/m^2^). Our basal depth estimates vary between 26 to 32 km for the southern Adriatic Sea basin and, toward the Ionian Sea basin, from 34 km (frontal wedge of the Calabrian arc) to 20 km (Calabrian accretionary wedge, where very low heat flow values (Fig. 7b, 30–40 mW/m^2^) have been attributed to an old oceanic crust^89^.

The Moho-depth model estimated from the vertical gradient of gravity data is shown in Fig. 7c. Similar to the depth to the bottom of magnetic sources model, the Moho boundary varies from about 11 km beneath the back-arc Tyrrhenian basin to more than 34 km the Apennines orogen.

We found that the Moho depth ranges from about 13 km in the offshore of western Italy progressively increasing toward the coast, to attain a depth of about 28 km. A maximum crustal thickness is inferred in the Apennines fold and thrust belt, with a maximum Moho depth beneath central Apennines, slightly deepening to about 32 km in the region around Mt. Vulture and to about 34 km below the Calabrian accretionary prism.

In the Apulian foreland region, the Moho depth ranges from 25 to 28 km. Our estimate agrees to a shallow Moho depth (27 km) estimated from seismic data^45,61,70^, which is believed to be caused by crustal uplift and magmatic intrusion. In the Calabrian arc, the Moho boundary gradually rises to about 30–32 km.

## Discussion

### Model of the carbonate basement

The definition of the Mesozoic carbonate platform in the Campanian region has been rapidly improved in time with a continuous availability of new geophysical and geological data. However, previous models of the carbonate basement are mostly based on irregularly distributed 2D cross-sections^3,4,11,28,90^ which cannot warrant a lateral continuity of interpretation. Similarly, well-log data only provide local information and with a limited depth extension. We have instead seen that gravity data inversion can successfully provide a continuous 3D depth model of the carbonate surface covering an area with regional extension. On the other hand, the depth resolution of gravity models is lower with respect to seismic and we need to assume valid constraints when perform the interface inversion.

**Figure 8:**
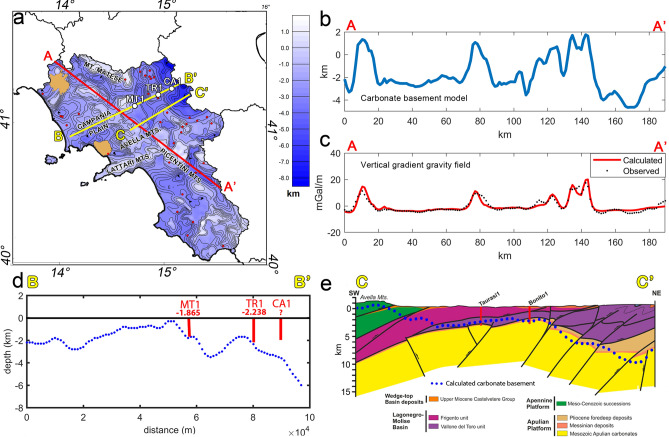
(**a**) Map of the carbonate basement with the plot of the selected profiles; (**b**) Morphology of the inferred carbonate basement model along the profile A-A′; (**c**) Fit of the vertical gradient calculated from the carbonate surface with the vertical gradient of the observed Free-air gravity field; (**d**) Comparison between the inferred carbonate model and the wells along the profile B-B′. MT1: MonteTaburno1 well; TR1: Tranfaglia1 well; CA1: Casalbore1 well. (**e**) Geological cross-section along the profile C–C′ (modified after^36^). The depths to the carbonate basement top estimated by gravity data inversion are drawn along the same profile (blue dots).

In order to validate our results, we calculated along the profile A-A′ (Fig. 8a) the vertical gradient due to the inferred carbonate surface and compared it to the observed data. We used the method by Parker^91^, which allows estimating the gravity field due to a layer with variable surface; as for the density contrast between the buried carbonates and the shallower sediments we assumed 0.4 g/cm^3^, while for the outcrop surface we set the average density of the Mesozoic carbonate units (2.6 g/cm^3^).

In Fig. 8b, we show the carbonate surface morphology along the same profile, ranging from 4.5 km depth to an altitude of around 1.7 km, corresponding to the top of the Picentini Mts.; finally, in Fig. 8c we compare the observed field of the Free-air vertical gradient (black dots) with the anomalies of the vertical gradient estimated from the carbonate surface (red line). The comparison shows that our model explains the observed gravity anomalies, so suggesting that the interface of the carbonate top represents by far the main source contribution to the free-air gravity field of the Campania region. A few misfits may be attributed to local heterogeneities and/or to the merging process between models of adjacent subareas.

To validate the inversion method, we excluded the wells MT1, TR1 and CA1 (white dots in Fig. 8a) from the set of constraints along the profile B-B′, and then compared the found model with the data of the 3 wells. This profile (Fig. 8d) is crossing the central part of the Campania region and the Apennine foredeep. The model of the carbonate basement shows depths ranging around 2 km beneath the Campania plain (western Campania), rapidly rising to shallow depths below Avella Mts. and progressively dipping toward the Bradanic Trough down to depths > 8 km (Fig. 8d). Our model well agrees with the boreholes MT1, TR1 where the Mesozoic carbonates was intercepted at depths of 1.866 km and 2.238 km, respectively. Similarly, we deduced a deep carbonate top (~ 3.8 km) below the CA1 well, extending down to 2.13 km without intercepting the carbonate.

A further comparison is shown in Fig. 8e with the geological section built by Vitale and Ciarcia^36^ along profile C-C′. The model shows the Apennine carbonate unit overlying the Lagonegro-Molise basinal units, and an Apulian carbonate basement deeply deformed below the foredeep and deepening westward beneath the Avella Mts. The inferred model of the Mesozoic platform is in good agreement with the geological section. In the western sector, our depth estimates correspond well with the outcrop carbonate unit of Avella Mts. and they progressively deepen eastward, reaching the average depths of the Apulian basement, in good agreement with the Taurasi1 and Bonito1 wells. The carbonate model is found to be deep (8–9 km depth) below the foredeep region and characterized by an anticline-like trend, according to the geological section^36^.

### Crystalline basement models

For the magnetic basement map, we found that the depth estimates are locally influenced by the extensive volcanic and magmatic rocks that characterize the Tyrrhenian region and the crust beneath the Mt. Vulture area. For this reason, the basement model deduced from the magnetic data analysis should not be considered as the surface of the crystalline basement (i.e., the bottom of carbonate rocks) but, instead, as the top of magnetic sources, whatever they could be (intra-sedimentary or intra-basement). Comparing our results with the results carried out by Cassano et al.^73^, we observe a general agreement in the Adriatic region where the basement ranges between 10 and 14 km depth and beneath the Calabrian arc, where the magnetic crust deepens abruptly down to 15 km. We found significant differences with the bottom of carbonate rocks in the middle of Southern Apennines, where our map shows a sharp uplift of the magnetic sources, reasonably associated to the intra-sedimentary volcanic rocks of the Mt. Vulture. A similar interpretation can be assumed along the Tyrrhenian coast of the Campania region, where the crust is deeply affected by the magmatism and volcanism of Mt. Vesuvius, Phlegrean fields and Roccamonfina. Instead, our results are in fair agreement with previous interpretations carried out along the 2D forward modelling^81^, crossing the Southern Apennines. Here the authors suggest a complex architecture of the lower crust made up of crustal wedges involving the magnetic basement, whose depths range between 10 and 15 km.

The crystalline basement model inferred from the gravity data analysis is almost in accordance with the average depths obtained from the magnetic anomalies, despite some discrepancies due to the presence of highly-magnetized rocks. A shallow crystalline depth is found beneath the Apulian foreland, as the result of crustal flexural tectonics, which rapidly deepens toward SW beneath the thrust front. Accordingly, the geological cross sections constructed along and across the Southern Apennines in the frame of the CROP project, identified a depth to basement at about 12 km beneath the Apennines fold and thrust belt fault, gradually decreasing to about 7 km beneath the Apulian foreland^3,4,23,37,92^. This prominent depression is also evident in several gravity modeling constructed across the Apennines and strongly reflects the shape of the gravity low observed in the Bouguer field map^75^. Finally, the shallow crystalline basement beneath the Tyrrhenian Sea is reasonably motivated by the general thin Quaternary and Plio-Quaternary sequences, as confirmed by seismic data interpretation^93,94^ and by significant tectonic stretching, which is manifested by several normal faults.

### Constraints on the lower crust through Moho boundary and bottom of magnetic source estimates

The obtained maps of the Moho boundary and the depth to the bottom of magnetic sources give useful constraints to interpret the overall limits of the lower crust and its main thermal features. The estimated crustal magnetic bottom reveals a variable thermal setting, strongly correlated to the structural and volcano-magmatic features and also with the trend of the heat flow values. In fact, the state at which rocks lose their ferromagnetic properties can be due to changes in lithological composition and/or temperature^95,96^. We in fact observed (“Depth to the bottom of magnetic sources and the Moho boundary” section) that the depth to the bottom of magnetic sources may not necessarily represent the Curie temperature isotherm. In very low heat-flow regions, *h*_*b*_ may correspond to the Moho rather than the actual Curie isotherm^97^. However, recent studies show that there are circumstances in which the upper mantle could also be magnetic^98^. Moreover, the Curie temperature depends on the magnetic mineralogy so that a Curie temperature surface may not be an isothermal surface and, in the presence of young volcanic rocks over thick sedimentary rocks, one may end up detecting the depth to the bottom of volcanic rocks which is not the Curie isotherm.

In our study region, there are zones where the depth to the bottom of magnetic source is considerably shallower than the Moho depth (i.e., Tyrrhenian Sea and volcanic provinces). These areas are also characterized by high heat flow values. Thus, it is more likely that these zones may represent Curie isothermal surface. On the other hand, the depth to the bottom of magnetic sources beneath the Apennines thrust and fold belts coincides with the estimated Moho depth and possesses similar trend. In this case, the magnetic bottom is most likely not the Curie isotherm, but the Moho boundary.

Regarding the Moho boundary, we observe, in general, a similar behavior to other models produced mainly from seismic studies^61,69,70^ and gravity^75–77^. Differences are found in some regions, such as beneath the Tyrrhenian crust. In this region, our estimated depths are around 11–12 km, while the Moho depth inferred from seismic data (Fig. 5.1 in Nicolich^69^; Fig. 10 in Artemieva and Thybo^70^) and previous studies based on gravity field inversion (Fig. 3 in Tassis et al.^77^) indicate a depth of about 10 km. The overall regional morphology is mostly consistent with other wide-angle seismic refraction/reflection data and with gravity modeling, where the Moho depth has been predicted to be deep throughout the Southern Apennines (30–40 km)^45,69–71,76,77^. Comparing our results with the Moho map proposed by Cassinis et al.^99^ we find a general accordance in the Adriatic region, where the Moho is estimated at around 26 km depth. Instead, below the accretionary prism, we estimated a sharp flexure of the Moho, similarly to the trend of the crystalline surface, which contrasts to the more gently dipping Moho boundary inferred by Cassinis et al.^99^.

### Matching models with 2D cross sections

**Figure 9:**
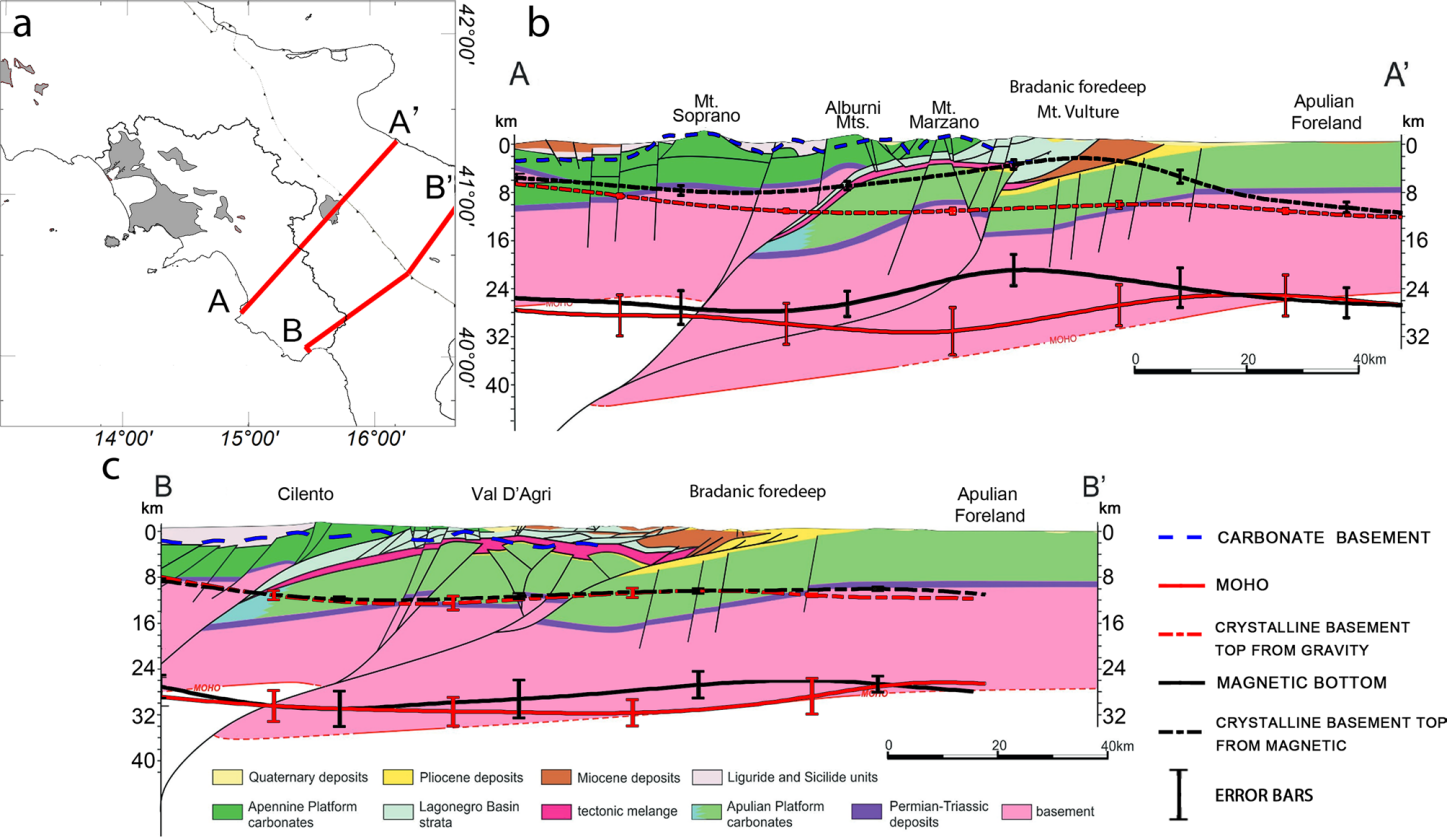
Crustal sections across the Southern Apennines (modified after^100^). Section A-A′ is along the trace of the CROP 04 deep seismic reflection profile. The carbonate basement top and spectral depth estimates are superimposed for comparison.

We further investigate the validity of our inferred depth models of the carbonate platform, crystalline basement, Moho and Curie isothermal surfaces comparing them to two geological sections^100^, mainly based on interpretation of seismic data (Fig. 9a), starting from the Cilento-Tyrrhenian region toward the Adriatic foreland. In particular, the cross-section A-A′ shown in Fig. 9 is drawn along the trace of the CROP-04 seismic reflection profile^101^. Both geological sections support a thick-skinned nature of the thrust-belt and associated crustal shortening, which implies the involvement of the deep crystalline basement into the crustal deformation and thrusting along a deep shear plane.

Our inferred carbonate basement is in good agreement with the trend of the Apennine carbonate platform along both profiles, showing very shallow depths beneath the Cilento and outcropping in correspondence with Mt. Soprano, the Alburni Mts. and Mt. Marzano (Fig. 9b,c). Along the A-A′ profile (Fig. 9b), the crystalline basement deduced from gravity data deepens smoothly from ~ 6 km beneath the Cilento to (~ 12 km) toward the foredeep area and then gently rises at around 10 km depth beneath the Apulian foreland. Therefore, even though the gravity basement resolution is not high (due to the window size of the spectral analysis) the average trend of the obtained crystalline surface could confirm a thick-skinned scenario of the crust below Southern Apennines. On the other hand, the depth to the top of the magnetic crust along the same profile is significantly different from that inferred from the gravity field, especially beneath the Bradanic foredeep. This is not surprising, since the profile crosses the volcanic region of the Mt. Vulture which, as explained above, inevitably affects the estimation of the top to the magnetic sources. Conversely, the comparison along the second profile B-B′ (Fig. 9c) shows a good agreement of both the depth to the magnetic top and the gravity crystalline basement with the geological model. We can in fact observe a very good match between the average depth of the top crystalline nappes below the Cilento area, the Val d’Agri and the Apulian foreland.

Regarding the Moho and bottom of the magnetic crust, we find that, apart from local discrepancies, the estimated depth has similar trend. However, along profile A-A′, we observe a shallower Moho below Mt. Marzano with respect to the geological model, reaching a maximum depth of around 32 km. Along profile B-B′, instead, our depth to the Moho boundary varies smoothly from around 27 km to the West to 33–34 km beneath the accretionary wedge of the orogen and progressively raises to 25 km beneath the Apulian foreland, in agreement with the seismic-interpreted Moho. In both cross-sections the depth to the bottom of magnetic sources is mostly consistent with the Moho depth, except below the Bradanic foredeep, along the profile A-A′ (Fig. 9b). We found there a rapid thinning of the magnetized crust which we may reasonably associate to the volcanic structure of Mt. Vulture volcano and/or to magmatic intrusions, as demonstrated by high heat flow values.

Although the inferred depth values are almost in agreement with the average shape of the seismic-interpreted Moho, our model does not clearly describe a mantle wedge in the lower crust^4,16,21,53,64,75,102^, while suggesting a lateral continuity of the crustal-mantle boundary from the Tyrrhenian crust to the Apulian foreland. However, we must consider that the inferred models provide a regional-scale interpretation of the deep structures, which may somewhere differ from local models from high-resolution data.

The standard errors of the cristallyne top depth varies along the selected profiles from about ± 0.1 km to about ± 1.5 km, being smaller beneath the Tyrrhenian Sea coastline and Apulian platform and larger beneath the Apennines thrust and fold belts. The standard error of the depth to the bottom of magnetic source along the seismic sections constructed across the Apennines ranges from about ± 2 km beneath the Tyrrhenian Sea coast to about ± 4 km beneath the Apennines thrust and fold belts. Similarly, the standard error of the depth to the Moho inferred from the vertical gradient of the gravity data varies from ± 2 km in the Tyrrhenian Sea coast to about ± 4.5 km in the Apennine chain. As expected, the uncertainty increases as the depth estimate increases.

We however note that our comparison with the depth estimated from seismic data is necessarily incomplete, because there is no information regarding the error estimation of seismic sections.

## Conclusions

Modelling of large-scale crustal structures is somewhat challenging along complex geological scenarios, which require broad integration of different geological and geophysical data. In this study we have shown that potential fields methods may represent an insightful and necessary tool to image of the subsurface geology, especially where direct information or other geophysical information is lacking. By these methods, we have modelled the crust of the Campanian region and Southern Apennines, a region where knowledge of the deep geological and geothermal setting has still open questions.

We adopted a stable and robust inverse method to map the complex morphology of the carbonate platform, which results in overall agreement with other sparse geophysical/geological information. The availability of detailed local information from wells and seismic data made possible to largely improve the model of the carbonate top, especially in areas where gravity inversion is unable to identify the boundary between the carbonate rocks and the overlaying geological units, having similar density.

Using spectral methods we describe a set of new maps of the Campanian region regarding the depth to the crystalline basement and Moho undulations (from the vertical gradient of gravity data) and the depth to the top and to the bottom of magnetic sources (from total field magnetic data). In fact, the depth to the crystalline basement beneath the Apennines belt is poorly known, as compared to other parts of the Italian Peninsula, due to lack of deep borehole data, poor seismic penetration, and geological complexity. Overall, our depth models of the crystalline surface inferred from gravity and magnetic spectral analysis show a variable morphology characterized by shallow depth beneath the Tyrrhenian back-arc basin, progressively increasing southward and toward the Apennines thrust and fold belt with more or less similar trend. In spite of local differences from the two estimated depth-models we show that the gravity-based model of the base of the carbonate layer is continuous and smoother than the magnetic basement, especially where there are extensive volcanic rocks on the surface or at shallow depth.

In the high heat-flow areas (i.e. Tyrrhenian Se and the magmatic provinces), the depths to the bottom of magnetic sources are significantly shallower than the inferred Moho depth, whereas in the Apennines thrust and fold belts the estimated depth to the bottom correlate with the Moho depth.

Although our models support the idea of thick-skin model, the nature and extent of crustal deformation in the deep crust is less likely to be inferred from this study. It is worthy to note that the depth to the bottom of magnetic sources, often interpreted as Curie isotherm, is an important constraint to develop thermal models on a regional scale. Likewise, the inferred model of the Mesozoic carbonate layer can be used to build the regional features of the low-to-medium enthalpy geothermal reservoirs.

## Methods

### Inversion for the carbonate basement top

Here we refer to the original paper by Fedi^84^. The inverse method used to model the carbonate surface top from gravity data consists of assuming an interface separating two media of different magnetization or density, which is modelled using a set of adjacent and homogeneous prisms with variable depth to the top and thickness.

The gravity field due to the effect of each prism (${G}_{ij}^{rs}$) can be expressed as:

1 $$\begin{aligned}{G}_{ij}&=\sum_{r=1}^{M}\sum_{s=1}^{N}{G}_{ij}^{rs}\left[v,{\left({h}_{p}\right)}_{rs},{\left({t}_{p}\right)}_{rs}, a, b, h, t\right] \\ & \quad i, r=\left\{1, \dots, M\right\}; \quad j, s=\left\{1, \dots, N\right\}.\end{aligned}$$

where ${G}_{ij}$ is the gravity field measurement of a discrete set of *M* × *N* data at points (${P}_{ij}$), with a step size of 2*a* and 2*b* along the *x* and *y* axes; *v* is the density (or magnetization) contrast between the prisms (*v*_1_) and the surrounding rock (*v*_*2*_); ${h}_{p}$ and ${t}_{p}$ are, respectively, the depth to the top and the thickness of a prism with constant horizontal size 2*a* × 2*b*. The inverse problem consists of determining ${h}_{p}$ and *v* from the data ${G}_{ij}$ assuming that ${\left({h}_{p}\right)}_{rs}$ can vary between *h* and *h* + *t*, which are fixed as constraints. Therefore, a-priori information is needed to constrain the shallowest depth to the top and the maximum thickness of the layer where the interface to find is contained.

By using such a-priori constraints, Eq. (1) can be expressed in terms of apparent densities ($\it {\Psi }_{ij}$) of an equivalent layer of depth *h* and thickness *t*:

2 $$\begin{aligned} {\Psi }_{ij}&=\sum_{r=1}^{M}\sum_{s=1}^{N}{\Psi }_{ij}^{rs}\left[v, {\left({h}_{p}\right)}_{rs}, {\left({t}_{p}\right)}_{rs}, a, b\right] \\ & \quad \quad i, r=\left\{1, \dots, M\right\}; \quad j,s=\left\{1, \dots, N\right\}.\end{aligned}$$

Therefore, the inverse problem here consists of inverting $\it {\Psi }_{ij}$ to find the prism thickness ${\left({t}_{p}\right)}_{ij}$, since they are linearly related to the depth to the top of the prism. The estimation of ${\left({h}_{p}\right)}_{rs}$ and *v* can be achieved by deriving the analytical expression of the Fourier transform of $\it {\Psi }_{ij}$:

3 $${\stackrel{\sim}{\Psi^{rs}}}\left(\alpha,\beta \right)=v{\stackrel{\sim}{\Phi^{rs}}}(\alpha,\beta)$$

where *α* and *β* are the wavenumbers and ${\stackrel{\sim }{\Phi^{rs}}}$ is the Fourier transform of the apparent density function of unit-density.

Then, the inverse Fourier transform of ${\stackrel{\sim }{\Phi^{rs}}}$ at the center of each prism is expressed by:

4 $${\it \Phi }^{ij}\left({x}_{i},{y}_{j}\right)=\frac{2}{\pi }\sum _{k=1}^{\infty }\left[{\tan}^{-1}\left(\frac{ab}{{t}_{k}\sqrt{{t}_{k}^{2}+{a}^{2}+{b}^{2}}}\right)-{\tan}^{-1}\left(\frac{ab}{{\eta }_{k}\sqrt{{\eta }_{k}^{2}+{a}^{2}+{b}^{2}}}\right)\right]$$

where

5 $$\begin{aligned}{t}_{k}&=kt-{\left({t}_{p}\right)}_{rs}-{w}_{rs}+{q}_{rs}\\ {\eta }_{k} &=kt-{w}_{rs}+{q}_{rs}\\ k & = \left\{1,\dots,\infty \right\}\end{aligned}$$

and ${w}_{rs}$ is the depth to the bottom while ${q}_{rs}$ is the observation point altitude, which is both provided as a-priori information. Thus, the forward problem can be finally expressed as:

6 $$\begin{aligned}{\Psi }_{ij}&=v \sum_{r=1}^{M}\sum_{s=1}^{N}{\Phi}_{ij}^{rs}\left[{\left({t}_{p}\right)}_{rs}\right] \\ & \quad \quad i,r=\left\{1,\dots,M\right\}; \quad j,s=\left\{1,\dots,N\right\}\end{aligned}$$

A non-linear inverse approach can be adopted to estimate the thickness ${\left({t}_{p}\right)}_{rs}$ of each prism by transforming the above system to a set of *M* × *N* independent equations. Then, Eq. (6) can be written as:

7 $$\begin{aligned}{\it \Psi}_{ij} &=v{\stackrel{-}{\lambda}}_{ij}{\stackrel{-}{\it \Phi_{ij}^{rs}}}\left[{\left({t}_{p}\right)}_{ij}\right] \\ & \quad \quad i=\left\{1,\dots,M\right\}; \quad j=\left\{1,\dots,N\right\}\end{aligned}$$

where ${\stackrel{-}{\Phi_{ij}^{rs}}}$ is the ‘thickness estimator’ and ${\stackrel{-}{\lambda }}_{ij}$ is the ‘similarity function’ representing the degree of correlation between $\it {\Psi }_{ij}$ and ${\stackrel{-}{\Phi_{ij}^{rs}}}$. Therefore, the thickness estimator is computed from Eq. (4) for a set of ${\left({t}_{p}\right)}_{ij}$ and the solution that satisfies the a-priori constraints is that producing the highest correlation between ${\stackrel{-}{\Phi_{ij}^{rs}}}$ and $\it {\Psi }_{ij}$*.*

### Spectral methods of potential field data for depth estimation

Different spectral techniques have been proposed for depth estimation of potential field data. Some of them assume a white noise source model^95,103–105^ others statistical source ensembles^85,106,107^ or a scaling/fractal source distribution^86,108–112^. The statistical block-ensemble source is suitable for homogeneous blocks of different sizes and magnetization (i.e., gross homogeneous bodies)^85,106^. The white noise is suitable for uncorrelated, highly variable magnetization/density distributions^103,113^, whereas the scaling source is suitable for describing irregular magnetization/density distributions which have however some degrees of correlation^114^.

Referring to the Spector and Grant’ model, using the statistical mechanics postulate, the mathematical expectation of an ensemble power density function is equal to the ensemble average^85^, so that the radial average power spectrum $\bar{E}(k)$ is:

8 $$\langle \bar{E}(k) \rangle=4{\pi }^{2}{\bar{M}}^{2} \langle{e}^{-2hk} \rangle \langle{T}^{2}(k) \rangle \langle{R}_{T}^{2} \rangle \langle{R}_{M^{2}} \rangle \langle{S}^{2}(k,a,b) \rangle$$

where *k* is the wavenumber, *R*_*T*_ is a factor related to the geomagnetic field direction, *R*_*M*_ is a factor related to the magnetization direction,${S}^{2}(k,\theta ,a,b)$ is a factor related to horizontal dimension sources (size factor). Spector and Grant^85^ observed that the size factor 〈*S*^2^(*k*, a, b)〉 could lead to an overestimation of the depth. However, they did not give any rule for correcting this effect and stated, mistakenly, that the factor had an exponential form. Fedi et al.^106^ showed instead that it is well approximated by a power-law form *k*^*β*^, with the decaying exponent of about − 2.9 (^106^, their Fig. 1). Therefore, provided the sources are very small, they proposed to correct the power spectrum by first dividing the spectrum by *k*^−β^ and then applying the Spector and Grant rule for depth estimation. With this correction, Eq. (8) becomes:

9 $$\mathit{ln}(\langle\bar{E}\left(k\right)\rangle)\approx \mathit{ln}(A)-2k\stackrel{-}{h}-\beta k$$

where $A=4{\pi }^{2}{\stackrel{-}{M^{2}}}\langle{R}_{T}^{2}\rangle \langle{R}_{M}^{2}\rangle$ is a constant, and $\beta =2.9$.

Since the vertical gradient of gravity is equivalent to a pseudomagnetic field at the pole, a simple correction to the gravity radially averaged power spectrum is required prior to making the slope-depth estimation. The correction is in forming the spectrum *kE*(*k*) which defines in the Fourier domain the pseudomagnetic transformation^115^. Equation (9) with *kE*(*k*) at the first member is then used, to estimate average depth to the top of magnetic and gravity sources from the slope of the power spectrum.

Regarding the depth to the source centroid, we assume the spectrum from fractal sources within a flat layer^86^:

10 $$E(k)=B{k}^{-\beta }{e}^{-2k{h}_{o}}{\left({e}^{-k({h}_{t}-{h}_{o})}-{e}^{-k({h}_{b}-{h}_{o})}\right)}^{2}$$

where $B=4\pi {C}_{m}^{2} \langle {R}_{T}^{2} \rangle \langle{R}_{M}^{2} \rangle$ is a constant, *h*_*t*_ is depth to the top, *h*_*o*_ the depth to the centroid, and $\beta$ is the fractal exponent. At long wavelengths, the estimate of the depth to the centroid is obtained from the slope of the radially averaged wavenumber-scaled power spectrum:

11 $$ln\left(\frac{E\left(k\right)}{{k}^{2}}\right)=lnC-2k{h}_{o}-\beta k$$

where $C=4\pi {C}_{m}^{2} \langle {R}_{T}^{2} \rangle\langle{R}_{M}^{2} \rangle {\Delta h}^{2}$. The depth to the bottom of the anomalous sources can then be easily computed as^103,116,117^:

12 $${h}_{b}=2{h}_{o}-{h}_{t}$$

In practice, spectral methods provide valid results if the optimal window size is chosen for the range of presumed depths^85,95^. In fact, the estimated depth change with the window size being shallower for smaller windows. In general, it is commonly accepted that large window sizes have the advantage to capture the longest wavelength; however, a large window size may lead to mix the content of different geological provinces and to give a general low resolution to the map of the estimated depths. In any case, there is no general agreement about the optimum window size.

Depending on the complexity of the study region and type of method adopted, a window size of 3 times the depth or more is generally used. The centroid and modified centroid methods require a window size of 3–5 times the maximum source depth^118^ the spectral peak method needs a window size of larger than 5 times the expected depth^95^. Only the nonlinear parameter estimation method^109^ requires an extremely large window size, say 10 times the expected depth^109,119^.

The standard error of the top and the centroid depths may be computed separately as^120^:

13 $$\varepsilon=\sqrt{\frac{1}{(n-2)}\left(\frac{{\sum}_{i=1}^{n}{\left({P}_{i}-{\widehat{P}}_{i}\right)}^{2}}{{\sum}_{i=1}^{n}{\left({k}_{i}-\bar{k}\right)}^{2}}\right)}$$

where $n$ is the number of observations, ${P}_{i}$ and ${\widehat{P}}_{i}$ are the observed and estimated values of the corrected power spectrum, ${k}_{i}$ and $\bar{k}$ are observed and mean values of the wavenumber considered for depth estimation. The standard error of the depth to the bottom of magnetic sources and Moho boundary may then be computed using:

14 $$\varepsilon=2{\varepsilon }_{o}+{\varepsilon }_{t},$$

where ${\varepsilon }_{o}$ and ${\varepsilon }_{t}$ are the standard errors of the centroid and top/crystalline depths, respectively^120,121^.

## Data Availability

The complete gravity and aeromagnetic datasets of Italy are available at the ISPRA website: http://portalesgi.isprambiente.it/en/elenco-base-dati/15. Heat flow data are available from the ‘Banca Dati Nazionale Geotermica del Consiglio Nazionale delle Ricerche’: http://geothopica.igg.cnr.it/. Well data are available at the ViDEPI website: https://www.videpi.com/videpi/videpi.asp.
